# Integrated Physiological and Biochemical Responses of Mungbean (*Vigna radiata*) to *Alternaria alternata*

**DOI:** 10.3390/plants15152397

**Published:** 2026-08-05

**Authors:** Dano Tillaboyeva, Hilola Matniyazova, Olga Myachina, Laziza Mavasalieva, Elena Abdrashitova, Gulrukhsor Ergasheva, Nodira Mamadalieva, Otabek Kuziev, Mingjigit Abdurakhimov, Dildora Amonova, Gulchexra Ashurova, Atanazar Rakhimov, Ramish Egamberdiev

**Affiliations:** 1 Laboratory of Ecological Physiology and Genetics of Industrial Crops, Institute of Genetics and Plant Experimental Biology, Academy of Sciences of the Republic of Uzbekistan, Tashkent 111208, Uzbekistan; 2 Faculty of Natural Sciences, Chirchik State Pedagogical University, Chirchik 111700, Uzbekistan; 3 Laboratory of Agrochemistry, Institute of General and Inorganic Chemistry, Academy of Sciences of the Republic of Uzbekistan, Tashkent 100170, Uzbekistan; 4 Faculty of Natural Sciences, National Pedagogical University of Uzbekistan Named After Nizami, Tashkent 100185, Uzbekistan; 5 Department of Medicine, Alfraganus University, Tashkent 100190, Uzbekistan; 6 Department of Soil Science and Agrotechnologies, Institute Agrobiotechnologies and Food Safety, Samarkand State University, Samarkand 140104, Uzbekistan; 7 Faculty of Natural Sciences and Agrobiotechnology, Bukhara State University, Bukhara 200100, Uzbekistan; 8 Department of Biology and Geography, Andijan State Pedagogical Institute, Andijan 170120, Uzbekistan; 9 Department of Soil Science and Agriculture, “Tashkent Institute of Irrigation and Agricultural Mechanization Engineers” National Research University, Tashkent 100000, Uzbekistan

**Keywords:** *Vigna radiata*, *Alternaria alternata*, salicylic acid, jasmonic acid, yield stability, photosynthetic pigments, water exchange

## Abstract

*Alternaria alternata* causes severe foliar disease and substantial yield losses in mung bean (Vigna radiata (L.) R. Wilczek). However, the physiological and hormonal mechanisms driving resistance in locally adapted varieties remain largely unknown. We evaluated nine genotypes of Uzbekistan under controlled *A. alternata* inoculation across four growth stages (budding, flowering, podding, harvest), measuring salicylic acid (SA), jasmonic acid (JA), photosynthetic pigments, leaf water relations, morphology, and yield components. Infection triggered highly variable SA responses. Surprisingly, strong SA induction offered no yield protection: Barqaror showed a 212% SA increase but suffered the most severe grain weight loss (−62.6%). Conversely, Turon maintained high baseline JA and was the only genotype to increase chlorophyll a at podding (+16.8%). Disease stress did not significantly affect 1000-seed weight (*p* = 0.184), indicating that *A. alternata* primarily disrupts early pod formation rather than the seed-filling process. Zilola demonstrated the best overall yield stability, exhibiting near-complete pod retention (−0.8%) and moderate grain weight loss (−26.7%) while maintaining acceptable seed size. These findings emphasize that protecting early pod set is more critical than seed-filling capacity under infection. Consequently, Zilola represents a highly promising parental line for breeding *A. alternata*-tolerant mung beans in Central Asia.

## 1. Introduction

Mung bean (*Vigna radiata* (L.) R. Wilczek) is one of the most widely grown grain legumes in Asia, valued both for its nutritional composition and for the agronomic services it provides to farming systems. The seed contains 24–28% protein alongside significant levels of carbohydrates, minerals, and vitamins, with the added advantage of producing less flatulence than most other legumes and having relatively low phytic acid content, which improves micronutrient bioavailability for human consumers [1,2]. Beyond its food value, mung bean fixes atmospheric nitrogen through symbiosis with *Rhizobium* spp., contributing up to 70 kg N ha^−1^ under effective nodulation and thereby reducing the fertiliser requirements of subsequent crops [3]. Its short growing cycle—typically 60–75 days to maturity—and tolerance of drought conditions make it particularly suitable for smallholder farming and for inclusion in crop rotation and intercropping systems across arid and semi-arid environments [4]. In Central Asia, including Uzbekistan, mung bean occupies an important niche as a subsistence and cash crop, and several locally adapted varieties have been developed and released for production under regional agroclimatic conditions [5,6].

Among the biotic constraints limiting mung bean yield, fungal diseases of the leaf and pod are among the most damaging. *Alternaria alternata* (Fr.) Keissl., the causal agent of Alternaria leaf spot, is a necrotrophic pathogen with a broad host range that causes brown-black necrotic lesions on leaves, dark discolouration of stems, and black spotting on pods, all of which reduce photosynthetic area, impair assimilate partitioning, and compromise seed quality [7,8]. Yield losses attributed to Alternaria leaf spot in susceptible mung bean cultivars have been reported to exceed 40% under conditions favourable for disease development [8,9,10]. The pathogen is particularly damaging during the vegetative and reproductive stages, when infection of foliar tissue coincides with active pod set and seed filling [8]. In Uzbekistan, both *A. alternata* and *A. tenuissima* have been documented under natural field conditions, causing necrosis on leaves of peas, soybeans, mung beans, and common beans, along with stem blight and pod spotting [11,12]. Despite this confirmed local presence, the response of Uzbek mung bean germplasm to *A. alternata* infection has not been systematically characterised.

Plant hormones—in particular salicylic acid (SA) and jasmonic acid (JA) play central roles in orchestrating defence responses to fungal pathogens. SA signalling is classically associated with systemic acquired resistance against biotrophic pathogens and is linked to the induction of pathogenesis-related (PR) proteins and reactive oxygen species production, though its role against necrotrophs is more variable and host-dependent [13,14]. JA-mediated pathways are generally considered more effective against necrotrophic fungi, activating defensive enzymes such as proteinase inhibitors and promoting cell wall reinforcement [15]. However, SA and JA frequently interact in an antagonistic manner: high SA accumulation can suppress JA-dependent defences, a dynamic that certain necrotrophic pathogens appear to exploit [16,17]. In *A. alternata* pathosystems specifically, this cross-talk has been shown to influence the outcome of infection in ways that are not easily predicted from single-hormone measurements [16,18]. In Arabidopsis, SA has been shown to suppress singlet oxygen (^1^O_2_)-mediated susceptibility to *A. alternata* by counteracting JA-dependent cell death signalling downstream of the EXECUTER 1/2 pathway [18], indicating that SA does not simply promote resistance but actively modulates the hormonal environment in which infection unfolds. The relative contributions of SA and JA to defence outcomes vary with genotype, growth stage, and infection intensity, making integrated multi-stage assessment necessary for any meaningful characterisation of resistance mechanisms.

Critically, hormonal induction reflects the initiation of a defence response rather than its effectiveness in protecting yield. Defence activation carries a metabolic cost, and resources allocated to signalling and defence compounds are unavailable for reproductive development [19,20]. Genotypes mounting the strongest hormonal responses therefore do not necessarily achieve the best agronomic outcomes, and hormonal metrics measured in isolation can mislead [21]. Physiological traits provide complementary predictive value because they capture downstream consequences rather than only the initial signalling event: pigment retention reflects carbon assimilation capacity, water relations indicate stomatal and membrane integrity, and morphological responses reveal assimilate partitioning between vegetative and reproductive sinks. Integrating the cumulative effects of infection over the season, these traits are more directly connected to final yield than single-timepoint hormonal measurements.

Previous work on mung bean responses to biotic stress has addressed individual aspects of pathogen interaction, including disease severity scoring, fungicide evaluation, and single-stage biochemical measurements [7,9,10]. When evaluating how plants respond to fungal pathogens, it is crucial to clearly separate “resistance” from “tolerance”—two completely distinct outcomes that are unfortunately often mixed up in disease studies. Essentially, resistance is the plant’s active ability to limit pathogen growth and spread. We typically measure this by observing lower disease severity scores compared to known susceptible plants [22]. Tolerance, on the other hand, means the plant can still function normally and produce a good yield even when heavily infected. Because visual disease symptoms do not tell the whole story here, tolerance is best measured by tracking the plant’s physiological health and final harvest data. Therefore, the most effective disease screening methods look at the whole picture. By combining visual disease scores with physiological and biochemical data, researchers can accurately distinguish the genotypes that actively restrict an infection from those that simply endure it [23]. Multivariate approaches—principal component analysis (PCA) and hierarchical clustering have been applied to mung bean germplasm evaluation under abiotic stresses such as drought and low phosphorus availability, successfully differentiating tolerant and sensitive accessions based on integrated physiological trait profiles [24,25]. However, an equivalent multi-trait, multi-stage analysis under *A. alternata* infection pressure has not been conducted for local mung bean germplasm. In particular, no study has simultaneously evaluated hormonal (SA and JA), physiological (photosynthetic pigments, water relations), morphological, and yield responses across the full crop cycle—from budding through to harvest—in varieties adapted to Central Asian growing conditions. This is a meaningful gap given that Uzbekistan has its own set of registered mung bean varieties whose disease response remains poorly understood and given the severity of Alternaria infections under local conditions [11,26,27].

The present study was therefore designed to address this gap. Nine locally registered mung bean genotypes were evaluated under control *A. alternata* inoculation across four developmental stages—budding, flowering, podding, and harvest. The work had four specific aims: to quantify SA and JA responses at the budding stage and relate these to subsequent physiological performance; to characterize genotype-specific changes in photosynthetic pigment content, leaf water relations, and morphological traits at the flowering and podding stages; to determine the effect of infection on yield components at harvest; and to integrate the resulting multi-trait data through PCA and hierarchical clustering to identify genotypes with superior yield stability and to define which trait combinations best distinguish tolerant from susceptible varieties. The findings are expected to support variety selection in disease-prone environments and to contribute a physiological and hormonal basis for future resistance breeding in this crop.

## 2. Results

### 2.1. Salicylic Acid and Jasmonic Acid Responses at the Budding Stage After 1 Dpi

**Figure 1:**
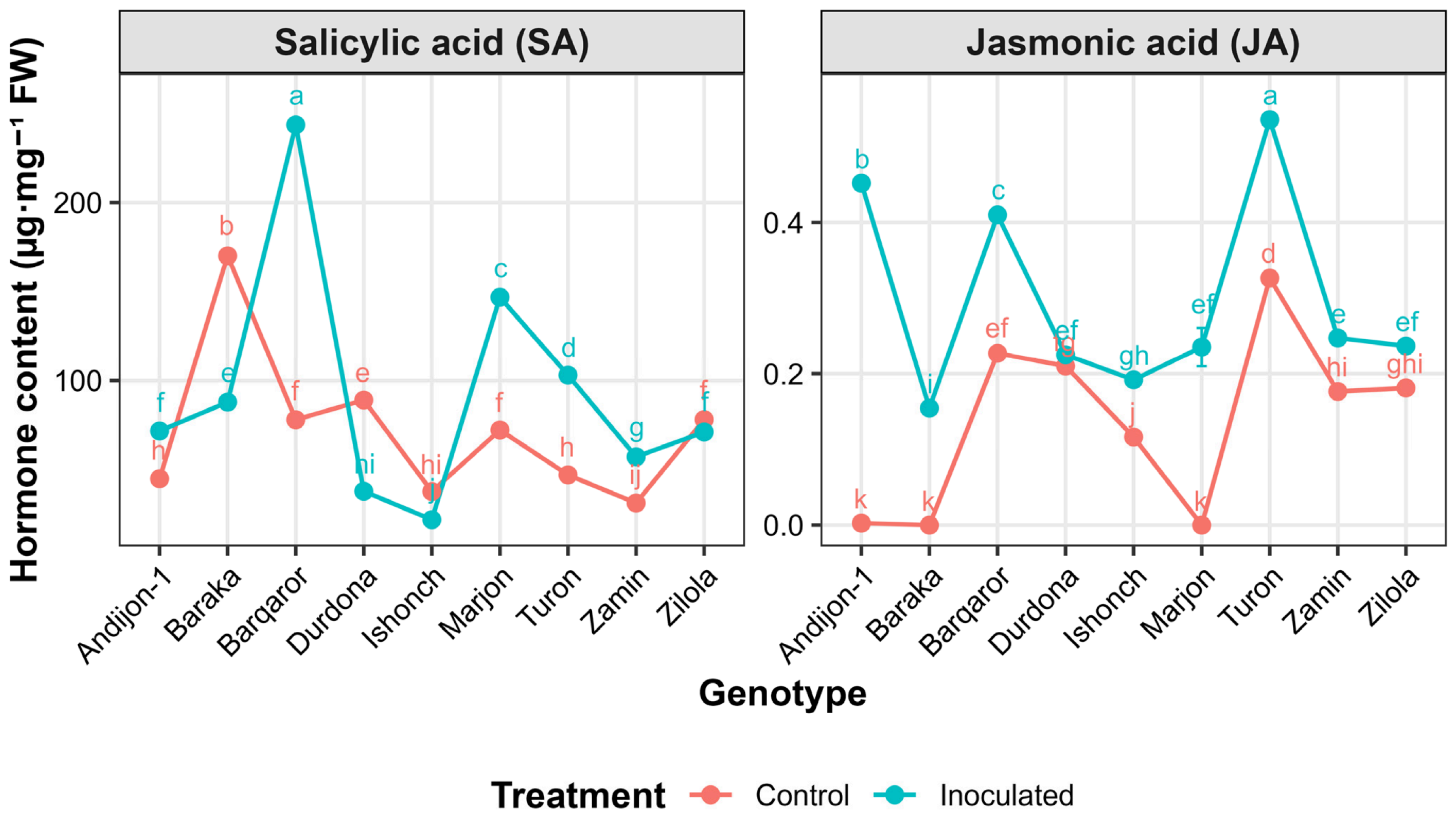
Salicylic acid (SA) and jasmonic acid (JA) contents (µg mg^−1^ FW) in nine mung bean (*Vigna radiata* L.) genotypes under Control and *A. alternata*-inoculated conditions at the Budding stage. Data points represent means ± SE (*n* = 3). Different lowercase letters indicate significant differences among genotype × treatment combinations (Tukey’s HSD, *p* < 0.05).

A significant effect of Genotype (G), Treatment (T), and their interaction (G × T) was detected for salicylic acid (SA) content at the budding stage (*p* < 0.001 for all sources). SA responses to *A. alternata* inoculation were strongly genotype-dependent, spanning from pronounced accumulation to marked decline (Figure 1). The largest increases were observed in Barqaror (+212.1%), Turon (+118.8%), Marjon (+103.1%), Andijon-1 (+59.6%) and Zamin (+82.6%), while SA declined in Baraka (−48.3%), Durdona (−57.4%), Ishonch (−41.8%), and Zilola (−8.9%). Baraka was notable for maintaining the highest basal SA concentration among all genotypes under Control conditions (170.17 µg mg^−1^ FW).

JA content was also significantly affected by Treatment and the G × T interaction (*p* < 0.001). Inoculation increased JA in all genotypes (Figure 1), though the magnitude of response varied considerably. The largest absolute increases were recorded in Andijon-1 (0.00 → 0.45 µg mg^−1^ FW) and Marjon (0.00 → 0.24 µg mg^−1^ FW). Turon was distinctive in exhibiting elevated JA under both Control (0.33 µg mg^−1^ FW) and Inoculated (0.54 µg mg^−1^ FW) conditions, while Zamin showed a more modest increase (0.18 → 0.25 µg mg^−1^ FW) and Durdona the smallest response overall (0.21 → 0.23 µg mg^−1^ FW).

### 2.2. Photosynthetic Pigment Contents at the Flowering and Podding Stages

#### 2.2.1. Pigment Content During Flowering Stage

Statistical analysis demonstrated significant effects of G (F = 7.288, *p* < 0.001), T (F = 61.475, *p* < 0.001), and G × T (F = 3.827, *p* = 0.002) on Chl *a* content at the Flowering stage (Table 1). Inoculation reduced Chl *a* in eight of nine genotypes; the greatest losses were recorded in Baraka (−35.6%), Zilola (−33.9%), and Zamin (−28.3%). Turon showed a negligible change (−1.3%), and Barqaror and Andijon-1 maintained Chl *a* with reductions of only 5.2% and 8.8%, respectively. Full mean values for all genotypes and treatments are presented in Table 1.

For Chl *b*, a significant interaction between G and T was not detected (*p* = 0.459), indicating a broadly consistent treatment effect across genotypes, though both main effects were significant (G: F = 2.355, *p* = 0.038; T: F = 8.903, *p* = 0.005). Carotenoid content showed a significant G × T interaction (F = 3.375, *p* = 0.005; Table 1), with Baraka recording the most pronounced decline and Barqaror maintaining the highest concentrations under inoculation.

#### 2.2.2. Pigment Content During Podding Stage

Pigment losses were larger at the Podding stage than at Flowering, and G × T interactions were significant for all measured pigments (Chl *a*: F = 14.934, *p* < 0.001; Chl *b*: F = 5.174, *p* < 0.001; Carotenoids: F = 8.730, *p* < 0.001; Table 2), indicating that genotypic differences in pigment response intensified as infection progressed.

The most severe Chl *a* reduction was recorded in Baraka (1.79 → 0.59 mg g^−1^ FW; −67.0%), followed by Durdona (−60.8%), Zilola (−59.9%), Barqaror (−59.5%), and Andijon-1 (−47.3%). Marjon and Ishonch showed intermediate reductions of −22.7% and −44.8%, respectively. Turon was the only genotype in which Chl *a* increased under inoculation (1.67 → 1.95 mg g^−1^ FW; +16.8%), and Zamin showed a small positive change (+3.7%; Table 2).

### 2.3. Leaf Water Relations at the Flowering and Podding Stages

#### 2.3.1. Leaf Water Relations at the Flowering Stage

Water content (WC) declined under inoculation in all genotypes at Flowering. The smallest reduction was in Turon (−1.3%; 76.07 → 75.07%) and the largest in Durdona (−9.5%; 78.81 → 71.30%).

**Figure 2:**
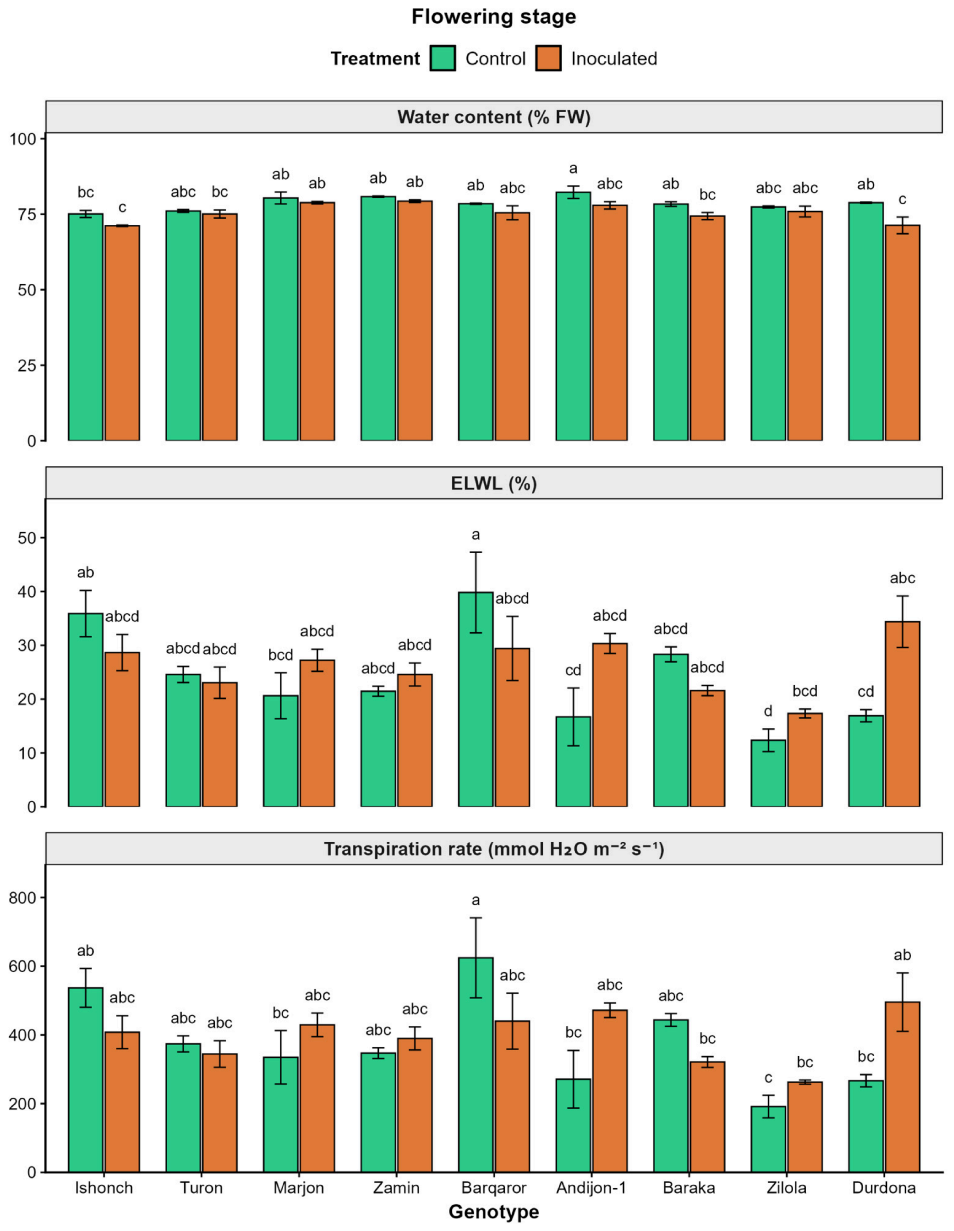
Leaf water content (WC, % FW), excised leaf water loss (ELWL, %), and transpiration rate (mmol H_2_O m^−2^ s^−1^) of nine mung bean (*Vigna radiata* L.) genotypes under Control and *A. alternata*-inoculated conditions at the Flowering stage. Bars represent means ± SE (*n* = 3). Different lowercase letters above bars indicate significant differences among genotype × treatment combinations (Tukey’s HSD, *p* < 0.05).

Excised leaf water loss (ELWL) and transpiration rate (TR) showed divergent responses between genotypes. ELWL increased in Andijon-1 (+13.6 percentage points, pp), Durdona (+17.5 pp), Marjon (+6.6 pp), Zamin (+3.1 pp), and Zilola (+5.0 pp), while decreasing in Ishonch (−7.2 pp), Baraka (−6.7 pp), and Barqaror (−10.4 pp). TR followed a broadly similar pattern: Durdona (+85.8%), Andijon-1 (+74.1%), and Zilola (+37.2%) showed the largest increases, while Barqaror (−29.5%), Baraka (−27.6%), and Ishonch (−24.0%) recorded the largest decreases. Turon showed the smallest TR reduction (−7.9%) and minimal ELWL change (−1.5 pp) among all genotypes (Figure 2).

#### 2.3.2. Leaf Water Relations at the Podding Stage

**Figure 3:**
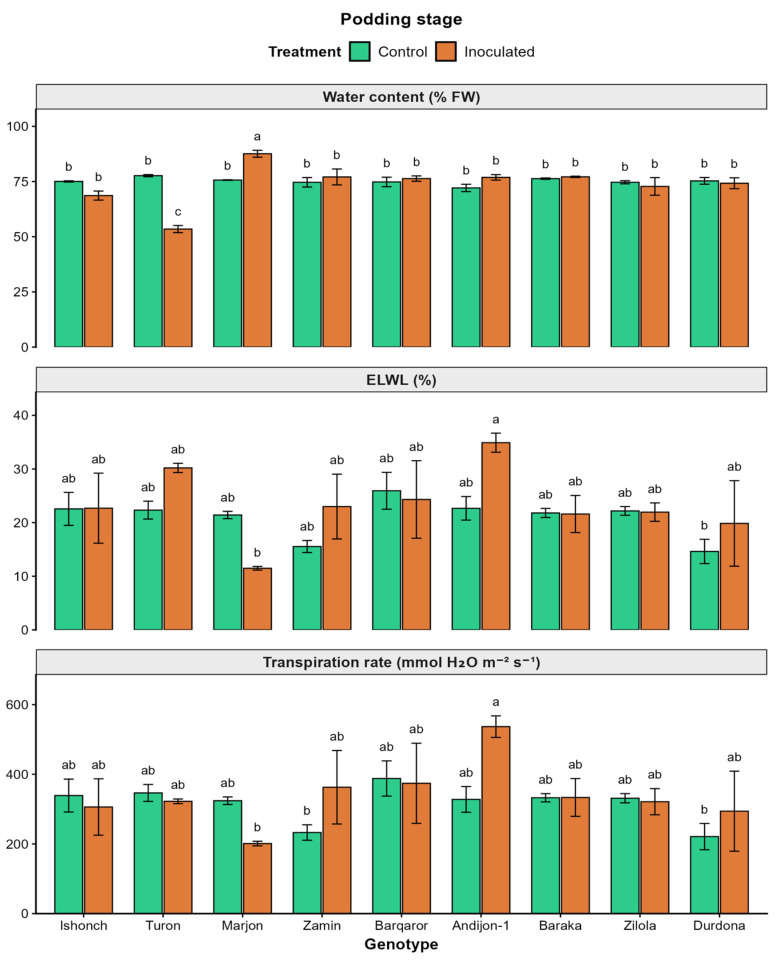
Leaf water content (WC, % FW), excised leaf water loss (ELWL, %), and transpiration rate (mmol H_2_O m^−2^ s^−1^) of nine mung bean (*Vigna radiata* L.) genotypes under Control and *A. alternata*-inoculated conditions at the Podding stage. Bars represent means ± SE (*n* = 3). Different lowercase letters above bars indicate significant differences among genotype × treatment combinations (Tukey’s HSD, *p* < 0.05).

WC responses at Podding differed notably from those at Flowering. Turon recorded the largest WC decline of all genotypes at this stage (77.67 → 53.47%; −31.2%). Five other genotypes maintained or slightly increased WC under inoculation—Marjon (+15.8%), Andijon-1 (+6.6%), Zamin (+3.2%), Barqaror (+2.0%), and Baraka (+1.1%), while Ishonch (−8.5%), Zilola (−2.5%), and Durdona (−1.4%) showed modest declines (Figure 3).

ELWL increased in Andijon-1 (+12.2 pp), Turon (+7.9 pp), and Zamin (+7.4 pp) at Podding, while Baraka, Zilola, and Barqaror showed negligible changes between Control and Inoculated conditions (Figure 3).

### 2.4. Morphological Traits at the Flowering and Podding Stages

#### 2.4.1. Morphological Traits at the Flowering Stage

A significant G × T interaction was observed for plant height (F = 7.337, *p* < 0.001), leaf number (F = 2.336, *p* = 0.039), and node number (F = 5.864, *p* < 0.001) at the Flowering stage (Table 3). Plant height responses varied substantially: Andijon-1 (+43.7%), Durdona (+35.1%), Marjon (+33.0%), and Zilola (+25.7%) showed increases under infection, whereas Barqaror (−26.0%) and Ishonch (−16.1%) showed reductions.

#### 2.4.2. Morphological Traits at the Podding Stage

At the Podding stage, inoculation reduced plant height in most genotypes. The largest decreases were in Durdona (−39.4%), Baraka (−31.9%), and Ishonch (−28.5%; Table 4). Zamin (−0.8%), Andijon-1 (−1.9%), and Barqaror (−6.6%) maintained heights closest to their respective Controls, while Turon showed a moderate reduction (−11.8%).

### 2.5. Yield Components at the Harvest Stage

#### 2.5.1. Pod Number and Pod Length

Treatment had a highly significant effect on pod number (F = 45.284, *p* < 0.001), and a significant G × T interaction was also detected (F = 2.213, *p* = 0.028), whereas the Genotype main effect did not reach significance (*p* = 0.067; Table 5). Pod number reductions ranged from −0.8% in Zilola (44.19 → 43.85) to −54.0% in Andijon-1 (68.60 → 31.56). Baraka recorded the second smallest reduction (−9.0%), while the largest losses were in Andijon-1 (−54.0%), Barqaror (−52.9%), and Zamin (−45.7%).

A significant G × T interaction was also observed for pod length (G: F = 7.855, *p* < 0.001; T: F = 85.864, *p* < 0.001; G × T: F = 2.267, *p* = 0.026). Reductions ranged from −7.4% in Barqaror to −33.6% in Baraka; Andijon-1 and Turon showed moderate decreases of −10.1% and −13.4%, respectively (Table 5).

#### 2.5.2. Grain Weight and 1000-Seed Weight

Grain weight declined significantly under inoculation in all genotypes (T: F = 74.051, *p* < 0.001; Table 5). Proportional losses ranged from −23.4% in Baraka and −26.7% in Zilola to −62.6% in Barqaror, with Zamin (−59.6%) and Durdona (−45.8%) also among the most severely affected. Baraka and Zilola retained the highest proportions of their yield potential under infection. However, it is worth mentioning that Baraka recorded the lowest absolute grain and seed weights in the trial: its 1000-seed weight under both Control (3.58 g) and Inoculated (3.03 g) conditions was approximately half that of Andijon-1 (7.77 g Control) and Zamin (7.18 g Control; Table 5).

In contrast to grain weight, 1000-seed weight showed no significant response to Treatment (F = 1.780, *p* = 0.184) or the G × T interaction (F = 0.611, *p* = 0.768). Only Genotype had a significant effect (F = 34.615, *p* < 0.001), with changes under inoculation ranging from −15.4% in Baraka to +5.3% in Ishonch across all genotypes. This finding indicates that individual seed-filling capacity is a stable, genotype-determined trait that remained largely unaffected by *A. alternata* infection, in contrast to pod number and total grain weight.

### 2.6. Multivariate Analysis

#### 2.6.1. Principal Component Analysis

**Figure 4:**
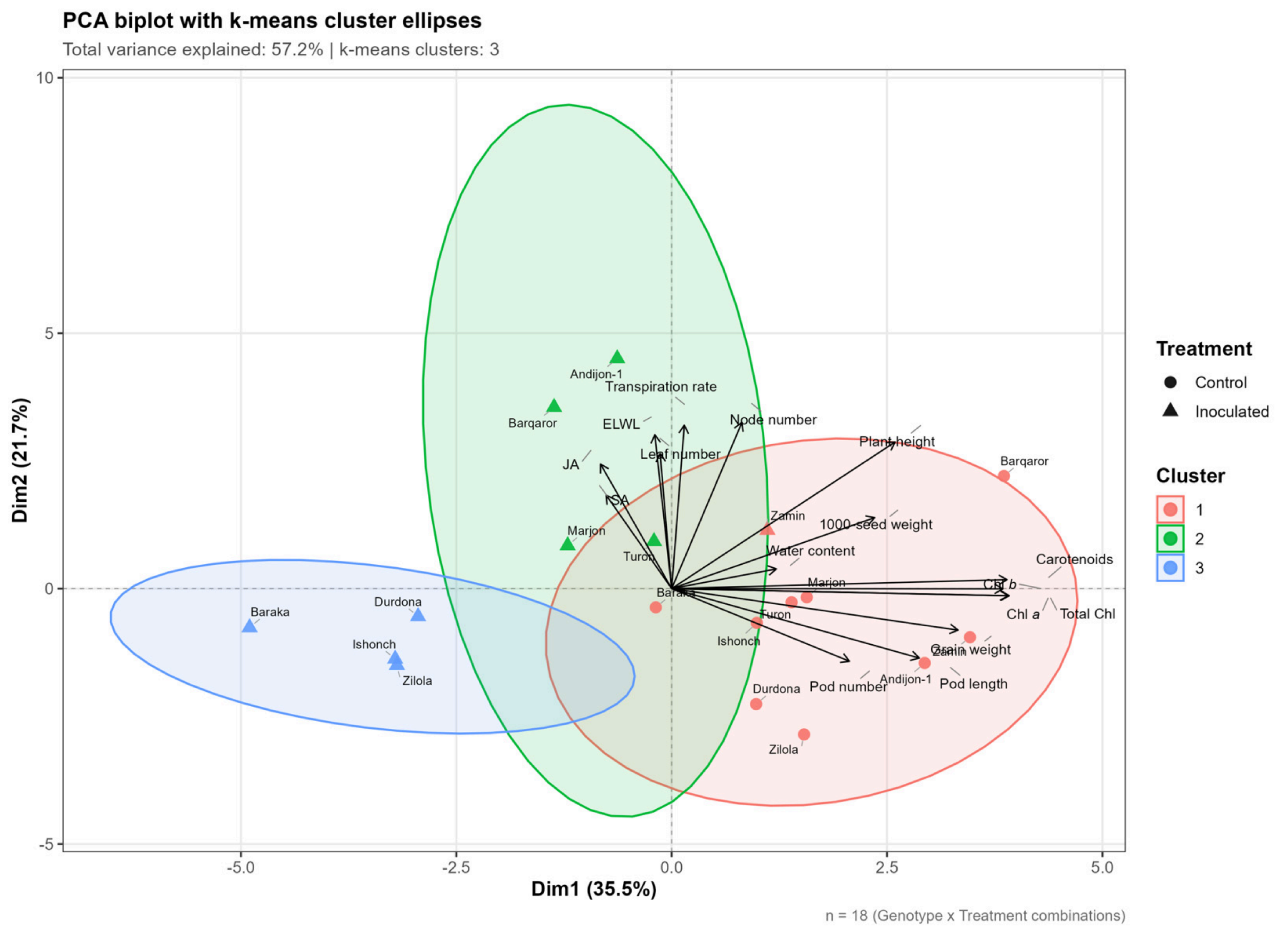
PCA biplot of 16 traits measured in nine mung bean (*Vigna radiata* L.) genotypes under Control (circles) and *A. alternata*-inoculated (triangles) conditions (*n* = 18 genotype × treatment combinations). Arrows indicate trait loadings on PC1 and PC2. Coloured ellipses represent k-means clusters (k = 3) at 95% confidence intervals. PC1 and PC2 collectively explained 57.2% of total variance.

PCA of the standardised genotype × treatment trait profiles (*n* = 18) explained 35.5% and 21.7% of total variance in PC1 and PC2, respectively, accounting for 57.2% in combination (Figure 4). PC3 contributed a further 12.5%, bringing the cumulative variance to 69.7% (Figure 4). PC1 was dominated by photosynthetic pigment traits (Chl *a*, Chl *b*, total chlorophyll, carotenoids; absolute loadings > 0.38) and total grain weight (loading: 0.33), with Control-treatment genotypes located in the positive PC1 region. PC2 was primarily associated with hormonal (SA, JA) and morphological (plant height, node number) traits.

K-means clustering (k = 3) identified three groups in PC1–PC2 space (Figure 4): Cluster 1 comprised predominantly Control-treatment genotypes with high pigment and yield values; Cluster 2 included inoculated genotypes retaining intermediate trait values, notably Turon and Zamin; and Cluster 3 consisted of inoculated genotypes with the largest infection-induced declines across traits. Control and Inoculated samples were clearly separated along PC1.

#### 2.6.2. Pearson Correlation Analysis

Pearson correlation analysis conducted separately for Control and Inoculated conditions revealed clear differences in inter-trait relationships between treatments (Supplementary Figure S1). Under Control conditions, Chl *a* and Chl *b* were strongly intercorrelated (r = 0.930, *p* < 0.001), as were Chl *a* and total chlorophyll (r = 0.986, *p* < 0.001). ELWL and transpiration rate showed a near-perfect correlation (r = 0.998, *p* < 0.001), and plant height was positively correlated with grain weight (r = 0.792, *p* = 0.011). Notably, SA was negatively correlated with grain weight (r = −0.772, *p* = 0.015) and 1000-seed weight (r = −0.860, *p* = 0.003) under Control conditions, indicating that genotypes with higher constitutive SA levels tended to produce lower seed yields in the absence of infection.

Under inoculation, chlorophyll intercorrelations were maintained (Chl *a*–Total Chl: r = 0.999, *p* < 0.001; Chl *a*–Chl *b*: r = 0.976, *p* < 0.001), and the ELWL–transpiration rate association remained strong (r = 0.902, *p* < 0.001). The positive correlation between plant height and grain weight observed under Control conditions was no longer significant under inoculation (r = 0.541, *p* = 0.133), reflecting the decoupling of vegetative growth from reproductive output under pathogen pressure. SA and JA showed no significant correlations with yield components under inoculation, consistent with the absence of a direct relationship between infection-induced hormonal responses and agronomic outcome.

#### 2.6.3. Hierarchical Clustering

**Figure 5:**
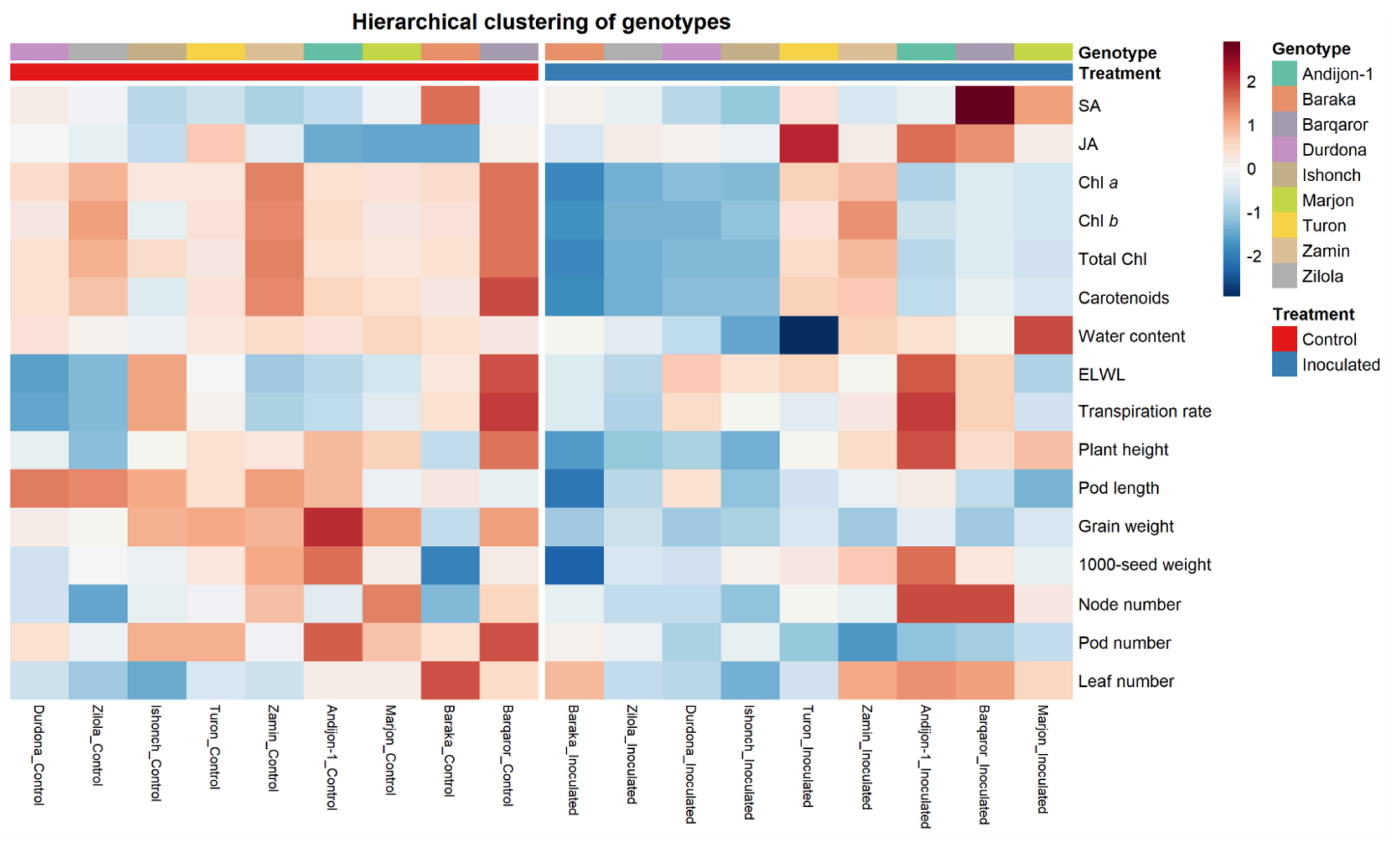
Hierarchical clustering heatmap of 16 standardised traits across nine mung bean (*Vigna radiata* L.) genotypes under Control and *A.alternata*-inoculated conditions (*n* = 18). Columns are grouped by Ward’s linkage with Euclidean distance. Colour gradient represents z-scores from negative (blue) to positive (red). Top bars indicate genotype identity and treatment.

Hierarchical clustering (Ward’s linkage, Euclidean distance) conducted separately within each treatment revealed substantial differences in genotype grouping between Control and Inoculated conditions (Figure 5). Under Control conditions, three clusters were identified: Cluster I (Durdona, Zilola; Euclidean distance ~2.1); Cluster II (Ishonch, Turon, Zamin, Andijon-1, Marjon); and Cluster III (Baraka, Barqaror, as separate outliers).

Under inoculation, the grouping structure changed substantially: Baraka, Zilola, Durdona, and Ishonch converged into a single cluster; Turon and Zamin formed a distinct pair; and Andijon-1, Barqaror, and Marjon constituted a third group. Genotypes previously grouped under Control conditions diverged under infection, while phenotypically dissimilar genotypes converged (Figure 5).

## 3. Discussion

### 3.1. Hormonal Defense Dynamics in Response to A. alternata

Effective defence against *A. alternata* begins with pathogen recognition, whereby fungal-derived molecular patterns are perceived by plant surface receptors, triggering an early signalling cascade that includes reactive oxygen species (ROS) production and activation of downstream hormonal pathways. This initial ROS burst serves a dual role: at controlled levels it functions as a signalling molecule that primes SA and JA biosynthesis, whereas excessive or sustained ROS accumulation—as induced by *A. alternata* toxins such as tenuazonic acid acting on the chloroplast electron transport chain—contributes to the oxidative damage underlying chlorophyll degradation and cell death observed in susceptible genotypes [28]. The genotype-specific outcomes reported here therefore likely reflect differences not only in hormonal output but also in the efficiency of ROS regulation and photosynthetic maintenance downstream of pathogen recognition, an interpretation consistent with the compensatory chlorophyll increase observed in Turon (Section 3.2) and the severe pigment degradation in Baraka and Barqaror.

The hormonal responses (SA and JA) to *A. alternata* inoculation were highly genotype-dependent, revealing that these mung bean varieties employ divergent biochemical strategies under identical pathogen pressure. The pronounced SA accumulation observed in Barqaror (+212%), Marjon (+103%), and Turon (+119%) aligns with typical SA-mediated defence mechanisms documented in legumes facing fungal stress [29]. Although SA signaling is primarily associated with systemic acquired resistance against biotrophic pathogens, its involvement in necrotrophic defense is distinctly complex [13,14]. Notably, the massive SA induction in Barqaror failed to confer yield protection; in fact, this genotype suffered the most severe reduction in grain weight (−62.6%). This suggests that SA-mediated defense is either ineffective against this pathogen or creates a severe physiological trade-off where the high energetic cost of producing SA limits reproductive development. Such a trade-off is consistent with the SA-JA antagonism observed in other pathosystems [16]. The molecular basis for this antagonism has been characterised in model plant species: when SA activates NPR1, it disrupts the interaction between MYC2 and MED25, which can silence JA-dependent gene expression even when JA levels are already high [16,17]. This exact mechanism has not yet been confirmed in mung bean, but it would account for the physiological patterns observed here. For the Barqaror genotype, the intense SA response may have restricted the JA-mediated defenses considered critical for defending against necrotrophs such as *A. alternata* [15], raising the possibility that effective resistance depends not just on how much hormone is produced, but on whether the functionally appropriate pathway, JA in this case, can operate without being overpowered by SA. Additionally, because SA and JA biosynthesis compete for shared metabolic resources, energy is inevitably diverted from plant growth, a defence–growth trade-off that could help explain why Barqaror’s substantial hormonal investment failed to protect its agronomic yield [19,20].

Conversely, Baraka exhibited the highest constitutive SA concentration prior to infection (170.17 µg mg^−1^ FW), followed by a notable post-inoculation decline (−48.3%). This dynamic may reflect a primed defensive state rather than an immunological failure: by downregulating active SA biosynthesis post-recognition, the plant could be prioritising response speed over continuous metabolic expenditure. Baraka’s relatively stable pod retention (−9.0%), compared with genotypes relying on intense post-infection SA bursts, fits with this interpretation [19,30].

Turon demonstrated an alternative strategy characterized by constitutively elevated JA levels (0.33 µg mg^−1^ FW in healthy plants) that increased moderately post-infection (0.54 µg mg^−1^ FW). Given that JA pathways are generally more effective against necrotrophs by activating proteinase inhibitors and defensive enzymes [15], Turon’s primed JA system theoretically offers rapid and broad-spectrum defense mobilization. However, this signaling did not entirely mitigate yield penalties (pod number decreased by 45.8%), indicating that JA priming alone is insufficient for complete tolerance. Nevertheless, this sustained JA activity may have facilitated Turon’s remarkable maintenance of Chl *a* during the podding stage (+16.8%), highlighting the crucial role of jasmonates in integrating defense responses with primary metabolism [31].

### 3.2. Photosynthetic Pigment Alterations Across Developmental Stages

The progressive reduction in chlorophyll content from flowering to podding across most genotypes is highly consistent with the necrotrophic lifestyle of *A. alternata*, which actively induces host cell death to extract nutrients. The pathogen secretes phytotoxins, such as alternariol and tenuazonic acid, known to compromise chloroplast membranes and inhibit photosystem II activity [28]. Consequently, the severe Chl *a* depletion observed in Baraka (−67.0%), Durdona (−60.8%), and Barqaror (−59.5%) at the podding stage indicates their limited capacity to protect photosynthetic apparatuses under continuous disease pressure.

Given that biotic stress conventionally suppresses photosynthesis-related gene expression [32], the notable +16.8% increase in Chl *a* observed in Turon during podding is highly atypical. This phenomenon likely reflects a compensatory mechanism: to maintain systemic carbon assimilation under sustained infection pressure, the plant upregulates chlorophyll biosynthesis in its uninfected leaf tissues. Zamin exhibited a similar directional trend (+3.7%), lending further support to this hypothesis, though the specific molecular basis driving this response requires future investigation.

Crucially, severe chlorophyll loss at podding did not strictly lead to final yield outcomes. For instance, despite experiencing the most drastic Chl *a* reduction, Baraka and Zilola retained the highest proportions of grain weight. In stark contrast, Barqaror suffered both severe pigment depletion and maximum yield loss. The absence of a clear relationship between mid-season chlorophyll loss and final grain yield suggests that mid-season chlorophyll content is not the sole determinant of productivity under *A. alternata* stress, pointing toward the critical role of efficient carbon remobilization from vegetative tissues to developing seeds.

### 3.3. Water Relations, Stomatal Dynamics, and Membrane Integrity

Leaf water status and stomatal behaviour varied considerably among genotypes in response to infection. The elevated transpiration and epidermal leaf water loss (ELWL) observed in Andijon-1 and Durdona at flowering likely stem from pathogen-induced stomatal dysfunction. Fungal pathogens are known to interfere with stomatal regulation through toxin-mediated disruption of guard cell signalling, thereby counteracting ABA-induced closure and promoting stomatal opening under infection [33]. While this inability to close stomata allows continuous gas exchange, it exacts a severe toll on tissue hydration, accelerating physiological decline under sustained infection.

In contrast, the reduced transpiration and ELWL in Baraka, Barqaror, and Ishonch are indicative of active stomatal immunity—a process where guard cells close rapidly upon detecting pathogen-associated molecular patterns (PAMPs) [34].

The substantial reduction in relative water content (RWC) in Turon during podding (−31.2%) presents an intriguing anomaly given its otherwise resilient physiological profile. This discrepancy might arise from the high metabolic water demand required to sustain simultaneous JA-driven defense responses and compensatory chlorophyll synthesis. Alternatively, cumulative fungal colonization by the podding stage may have compromised cellular membrane integrity despite the preservation of photosynthetic pigments, resolving this question will require more frequent sampling within the podding stage in future work.

### 3.4. Pathogen-Induced Morphological Modifications

The significant increases in plant height among Andijon-1 (+43.7%), Durdona (+35.1%), Marjon (+33.0%), and Zilola (+25.7%) at the flowering stage present an interesting observation that warrants careful interpretation. Paradoxical pathogen-induced growth stimulation has been documented in various plant–pathogen interactions, where infection disrupts the normal balance of phytohormone signalling in ways that transiently promote vegetative growth at the expense of reproductive development [19]. The concurrent severe losses in pod number and grain weight in Andijon-1, Durdona and Marjon at harvest are consistent with this interpretation—whatever the underlying mechanism, infection-associated vegetative stimulation at flowering appears to have come at a direct cost to reproductive output.

By the podding stage, this stimulation had reversed in most genotypes, giving way to general growth suppression reflecting progressive resource depletion under sustained infection. This transition aligns with jasmonate-mediated growth suppression documented under prolonged biotic stress [19], where sustained defence activation increasingly diverts resources away from growth. Zamin (−0.8%) and Andijon-1 (−1.9%) were notable exceptions, maintaining plant height close to their respective controls at podding, indicating sustained vegetative growth capacity under advanced infection.

### 3.5. Yield Component Impacts and Agronomic Tolerance Assessment

As explained in Section 2.4, we did not quantitatively score disease severity in this study. The genotype rankings presented below therefore reflect tolerance, meaning the plant’s ability to maintain yield despite infection, rather than true resistance, which refers to the plant’s capacity to restrict pathogen growth. Studies that quantify resistance typically combine standardised disease severity scales with physiological correlates [22], an approach that was not available in the present dataset. The genotypes exhibiting the most pronounced infection-induced height increases at flowering, namely Andijon-1, Durdona, and Marjon, also showed substantial pod number and grain weight losses at harvest [21], underscoring that morphological responses to infection at earlier growth stages can have lasting consequences for final yield.

The profound impact of *A. alternata* on grain weight across all genotypes, with reductions spanning 23% to 63%, reflects substantial genetic variability within the tested germplasm for yield stability under disease pressure. Such variability is a prerequisite for progress through targeted breeding [21], and the range observed here suggests that meaningful gains in tolerance are achievable without resorting to exotic germplasm.

Pod number emerged as the most volatile yet agronomically critical yield determinant in this study. Zilola demonstrated exceptional resilience, maintaining near-complete pod retention (−0.8%), whereas most other genotypes lost between 27% and 54% of their pods. Notably, Zilola also showed a modest height increase at flowering (+25.7%), yet unlike Andijon-1, Durdona, and Marjon, which exhibited similar or greater vegetative stimulation, this did not translate into substantial reproductive losses, suggesting that Zilola may possess a superior capacity to partition assimilates toward developing pods even under conditions of increased vegetative demand. The mechanism underlying this stability remains to be established; sustained assimilate partitioning toward reproductive sinks during early pod initiation has been identified as a key determinant of pod retention in legumes under stress [35], and this may represent one contributing factor in Zilola. Coupled with a commercially acceptable 1000-seed weight that declined only marginally under inoculation (5.85 → 5.32 g), Zilola stands out as the most agronomically relevant tolerant genotype identified in this study.

Baraka requires a more nuanced evaluation. While it exhibited the smallest proportional grain weight reduction (−23.4%) and strong pod retention (−9.0%), its absolute 1000-seed weight (3.58 g) falls well below commercially acceptable thresholds for Central Asian markets, where varieties such as Andijon-1 (7.77 g) and Zamin (7.18 g) set a higher baseline. A variety that retains a larger proportion of an already low yield is not agronomically equivalent to one that protects a substantially higher productive capacity. Baraka’s constitutive SA-based defence characteristics make it an interesting genetic resource, but these traits alone are insufficient to recommend it for direct deployment in production systems.

The statistically non-significant effect of inoculation on 1000-seed weight across the entire panel (F = 1.780, *p* = 0.184) is one of the more practically informative findings of this study. It indicates that *A. alternata* pathogenesis primarily disrupts early pod set and overall biomass accumulation, rather than the grain-filling process itself once pods are successfully initiated. This pattern has a direct implication for breeding strategy: protecting floral and early pod development stages, rather than improving individual seed-filling capacity—should be the primary target for *A. alternata* tolerance improvement in mung bean.

Finally, Barqaror provides perhaps the clearest illustration in this dataset of how defensive investment and agronomic outcome can diverge. The strongest SA response in the trial (+212%) was associated with the largest grain weight loss (−62.6%) and among the greatest pod number reductions (−52.9%). This outcome is consistent with the yield penalty hypothesis of constitutive resistance, which proposes that genotypes allocating substantial resources to defence mechanisms incur productivity costs that are not offset by protection gains under certain pathogen interactions [19,20]. This direct link between SA levels and reduced growth has been clearly demonstrated before. For instance, in transgenic *Arabidopsis* plants engineered to produce different amounts of SA, both biomass and seed yield dropped as total SA increased [36]. Even though that specific study did not involve a pathogen, it demonstrates that accumulating SA costs the plant valuable resources and limits its development, regardless of the trigger. This is consistent with our findings for the Barqaror genotype, where a strong SA response was paired with a severe loss in yield. It also reinforces the point that SA–JA antagonism, discussed in Section 3.1, may have further undermined the effectiveness of Barqaror’s primary defence investment. Taken together, these results demonstrate that hormonal defence metrics in isolation are unreliable proxies for agronomic tolerance and must always be evaluated alongside yield component data. Other studies have reported similar genotype-specific differences in both defense responses and post-infection performance across various crops [37]. This illustrates that evaluating multiple traits simultaneously is an effective and broadly applicable strategy for identifying disease-tolerant plants.

### 3.6. Multivariate Analysis of Defense and Yield Traits

Principal component analysis and hierarchical clustering provided a systematic, trait-integrated perspective on the phenotypic shifts induced by *A. alternata* infection that complements and extends the univariate analyses presented in previous sections. The distinct separation of Control and Inoculated groups along PC1, driven primarily by photosynthetic pigment content and yield-related traits, indicates that pathogen infection induced a consistent, reproducible shift in the overall physiological profile of all nine genotypes rather than isolated effects on individual traits. The 57.2% of total variance explained by PC1 and PC2 combined indicates that the dominant axes of phenotypic variation in this dataset are biologically interpretable rather than noise-dominated.

The k-means clustering (k = 3) superimposed on the PC1–PC2 ordination space identified three functionally coherent groups: a high-performance cluster comprising predominantly Control-treatment genotypes, an intermediate cluster of inoculated genotypes that maintained partial physiological capacity (notably Turon and Zamin, consistent with their chlorophyll maintenance documented in Section 3.2), and a third cluster of inoculated genotypes showing the most pronounced multi-trait decline. The consistency between these data-driven groupings and the univariate Tukey HSD groupings reported in Section 3.2, Section 3.3, Section 3.4 and Section 3.5 strengthens confidence in both analytical approaches and suggests that the major patterns of genotype differentiation under *A. alternata* stress are robust.

The restructuring of hierarchical clustering topology between Control and Inoculated conditions was equally informative. Under Control conditions, clustering largely reflected baseline physiological vigour, with Baraka and Barqaror emerging as outliers due to their atypical constitutive SA levels and divergent baseline trait profiles (Section 3.1 and Section 3.5). Under inoculation, this structure was substantially rearranged: Baraka converged with Zilola, Durdona, and Ishonch—genotypes with which it shared little phenotypic similarity under Control conditions—reflecting a convergence of stress-response profiles characterised by severe pigment degradation alongside relatively stable seed weight. Turon and Zamin formed a distinct pair defined by their shared capacity for chlorophyll maintenance under infection. These rearrangements illustrate that pathogen stress reveals a different landscape of genotypic similarity from that observed under optimal conditions—a finding with direct practical implications for germplasm screening.

The positive correlation between plant height and grain weight observed under Control conditions (r = 0.792, *p* < 0.05) deteriorated markedly under inoculation, consistent with the well-documented decoupling of vegetative vigour from yield performance under biotic stress. This decoupling represents a fundamental challenge for breeding programmes: phenotypic evaluation under favourable conditions can inadvertently select against, or simply fail to identify, genotypes with superior disease-associated yield stability [21,38]. The present results strongly support the necessity of evaluating mung bean germplasm directly under *A. alternata* infection pressure rather than relying on proxy traits measured under disease-free conditions.

## 4. Materials and Methods

### 4.1. Experimental Site and Plant Material

Scientific research was conducted during the 2025 growing season under lysimeter and controlled laboratory conditions at the experimental field of the Regional Experimental Station of the Institute of Genetics and Plant Experimental Biology, Academy of Sciences of the Republic of Uzbekistan. Lysimeter conditions allowed full monitoring and control of soil water parameters throughout the growing season, maintaining optimal moisture levels for plant growth with natural photoperiod conditions.

The experimental material comprised nine locally registered mung bean (*Vigna radiata* (L.) R. Wilczek) genotypes: Ishonch, Turon, Marjon, Zamin, Barqaror, Andijon-1, Baraka, Zilola, and Durdona. All varieties were obtained from the seed collection of the Research Institute of Plant Genetic Resources of the Republic of Uzbekistan.

### 4.2. Pathogen Culture and Inoculum Preparation

An isolate of *Alternaria alternata* (Fr.) Keissl. was obtained from the Unique Scientific Fund “Collection of Phytopathogenic and Other Microorganisms” maintained at the Institute of Genetics and Plant Experimental Biology, Academy of Sciences of the Republic of Uzbekistan. Two inoculum preparation protocols were applied depending on the experimental objective, as detailed below.

For lysimeter-based experiments assessing physiological, morphological, and yield-related traits, the pathogen was cultured in 100 mL of Czapek-Dox liquid medium in 250 mL Erlenmeyer flasks for 15 days at 25–27 °C with continuous shaking [39]. The resulting culture suspension was filtered through sterile gauze, and the conidial concentration was determined using a Goryayev hemocytometer and adjusted to 1 × 10^5^ conidia mL^−1^ with sterile distilled water. Czapek-Dox medium was selected for this purpose as it supports abundant fungal biomass production suitable for large-scale inoculation of lysimeter-grown plants.

For phytotron-based experiments assessing phytohormone content, the isolate was cultured on carrot agar medium for seven days at 25 °C [40]. Carrot agar was selected because it promotes profuse conidiation in *Alternaria* species, producing a uniform, high-titre conidial suspension. Conidia were washed from the agar surface with sterile distilled water, counted using a Goryayev hemocytometer, and adjusted to 1 × 10^6^ conidia mL^−1^.

A completely randomised design was used with two treatment blocks: (1) Control—plants grown under optimal conditions without pathogen exposure; and (2) Inoculated—plants subjected to artificial infection with *A. alternata*. Inoculation was performed at the vegetative growth stage by spray application of the prepared conidial suspension (1 × 10^5^ conidia mL^−1^) to the point of runoff on all above-ground plant surfaces. Control plants were sprayed with an equal volume of sterile distilled water. Biological replicates consisted of three independently grown plants per genotype per treatment for biochemical and physiological traits, and five plants per genotype per treatment for morphological measurements.

### 4.3. Pathogenicity and Disease Confirmation

**Figure 6:**
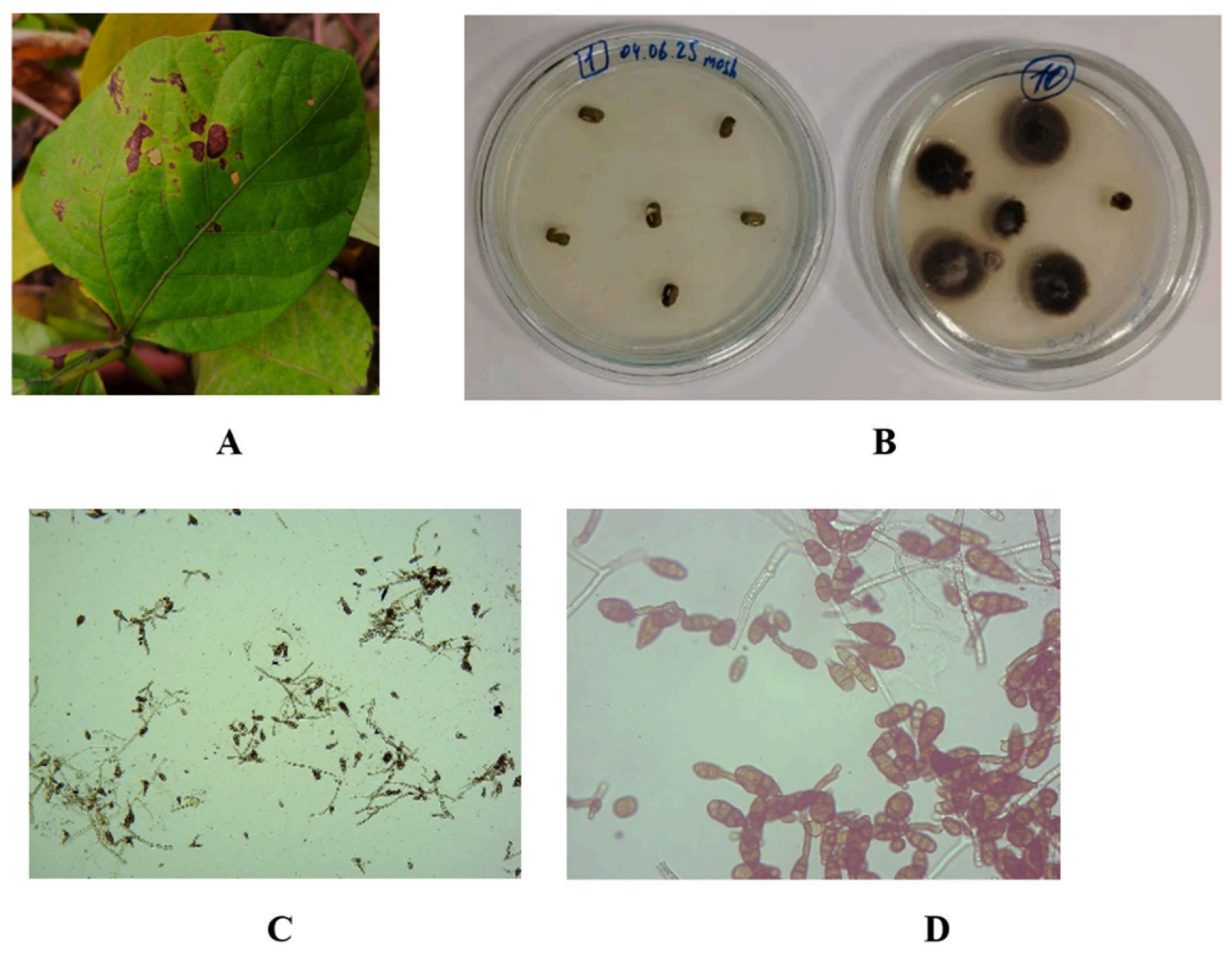
Confirmation of *A. alternata* pathogenicity on mung bean (*Vigna radiata* L.). (**A**) Characteristic necrotic lesions on a mung bean leaf following artificial inoculation; (**B**) potato dextrose agar (PDA) plates showing fungal colonies re-isolated from inoculated (**right**) and control (**left**) harvested seeds after 7 days of incubation at 25 °C; (**C**) conidiophores and conidia of *A. alternata* under light microscopy (×100); (**D**) characteristic multicellular conidia showing transverse and longitudinal septa (×400), confirming pathogen identity in accordance with Koch’s postulates.

Disease development in inoculated plants was monitored visually throughout the experiment. Characteristic brown-black necrotic lesions appeared on leaf surfaces of all inoculated plants within 7–10 days post-inoculation (dpi), and were absent in all Control plants (Figure 6A). To confirm pathogen identity following the experiment, seeds harvested from symptomatic inoculated plants were surface-sterilised with 1% sodium hypochlorite solution for 3 min, rinsed three times with sterile distilled water, and plated on potato dextrose agar (PDA). After seven days of incubation at 25 °C, fungal colonies were recovered from inoculated seeds but not from control seeds (Figure 6B), and confirmed as *A. alternata* based on colony morphology and microscopic examination of conidiophores and conidia (Figure 6C,D) [41]. This re-isolation served as qualitative confirmation of pathogen identity in accordance with Koch’s postulates.

### 4.4. Determination of Salicylic and Jasmonic Acids

Leaf samples for phytohormone analysis were collected at 1 day post-inoculation (1 dpi) during the Budding stage from the phytotron experiment. This early timepoint was chosen to capture the initial hormonal signalling response to pathogen recognition, before later physiological and developmental changes could obscure the signal. Salicylic acid (SA) and jasmonic acid (JA) contents were determined by high-performance liquid chromatography (HPLC) using an Agilent 1100 series system (Agilent Technologies, Santa Clara, CA, USA) equipped with a G1322A degasser, a binary pump, and a Zorbax SB-C18 reversed-phase analytical column (150 × 3.0 mm, 3.5 µm particle size) maintained at 30 °C.

For SA determination, 2.0 g of fresh leaf tissue was extracted in 30 mL of 70% ethanol at 80 °C for 30 min in a water bath, followed by centrifugation at 4000 rpm. To release bound salicylates, the extract was hydrolysed with 10 mL of 2 M hydrochloric acid at 80 °C for 60 min, then centrifuged again at 4000 rpm. The final extract was filtered through a 0.45 µm membrane filter prior to injection. The mobile phase consisted of 0.1% (*v*/*v*) orthophosphoric acid in water (25%), distilled water (25%), and acetonitrile (50%), delivered at a flow rate of 0.75 mL min^−1^ with an injection volume of 10 µL. Detection was performed using a fluorescence detector at excitation and emission wavelengths of 310 nm and 450 nm, respectively. Quantification was performed using an external standard calibration curve, and SA content was expressed as µg mg^−1^ fresh weight (FW) [42].

For JA determination, 1.0 g of fresh leaf tissue was extracted in 10 mL of methanol/water/acetic acid (80:19:1, *v*/*v*/*v*) with ultrasonic extraction at 15 °C for 30 min. The extract was filtered, and the residue was re-extracted once under the same conditions. Combined extracts were centrifuged at 12,000 rpm, and the supernatant was filtered through a 0.45 µm syringe filter prior to analysis. Separation was performed on a Perkin Elmer C18 column (250 × 4.6 mm, 5 µm) with an Agilent Zorbax guard cartridge, using a gradient mobile phase of 0.5% acetic acid in water and acetonitrile at a flow rate of 0.75 mL min^−1^, column temperature of 33 °C, and UV detection at 210 nm. JA content was expressed as µg mg^−1^ FW. This HPLC-UV method for JA quantification was optimised specifically for this study, adapting standard reversed-phase chromatographic principles to the extraction and detection conditions described above.

### 4.5. Photosynthetic Pigment Content

Leaf samples for pigment analysis were collected at the Flowering and Podding stages from the third and fourth leaves from the apical meristem. For each biological replicate, 50 mg of fresh leaf tissue was collected from a single randomly selected plant per genotype per treatment and homogenised in 10 mL of 95% ethanol. Three biological replicates (three independent plants) were analysed per genotype per treatment at each growth stage. The absorbance of the supernatant was measured using a UV–Vis spectrophotometer (Agilent Technologies, Santa Clara, CA, USA) at 664, 649, and 470 nm, corresponding to chlorophyll *a* (Chl *a*), chlorophyll *b* (Chl *b*), and carotenoids, respectively. Pigment concentrations (mg g^−1^ FW) were calculated using the equations of Nayek et al. (2014) [43]:

Chl *a* = 13.36 A664 − 5.19 A649

Chl *b* = 27.43 A649 − 8.12 A664

Carotenoids = (1000 A470 − 2.13 Chl *a* − 97.63 Chl *b*)/209

Total chlorophyll was calculated as the sum of Chl *a* and Chl *b*. Three biological replicates were analysed per genotype per treatment at each growth stage.

### 4.6. Water Exchange

Water content (WC), excised leaf water loss (ELWL), and transpiration rate (TR) were measured at the flowering and podding stages using the second and third leaves from the apical meristem. Three biological replicates per genotype per treatment were analysed at each stage.

WC was determined gravimetrically following Tretyakov et al. [44]. Fresh leaf samples (minimum 5 g per replicate) were placed in pre-dried and pre-weighed aluminium containers, weighed immediately (fresh weight, FW), and dried at 105 °C until constant mass was achieved (dry weight, DW). RWC was calculated as:

WC (%) = [(FW − DW)/FW] × 100

ELWL was assessed following Farshadfar et al. [45]. Freshly excised youngest leaves were weighed (FW), left at ambient conditions for 2 h, then wilted at 35 °C for a further 2 h and reweighed (wilted weight, WW_2h_). ELWL was calculated as:

ELWL (%) = [1 − (FW − WW_2h_)/FW] × 100

TR was determined following Ivanov et al. [46]. Freshly excised leaves (400–500 mg) were placed on a calibrated analytical balance, and mass was recorded at 1–2 min intervals for up to 15 min after excision. Leaf area was traced on paper and measured in cm^2^. TR was calculated as:

TR = (A × 60 × 1000)/(B × S) × 100

where A is the decrease in leaf mass (g), B is time (min), and S is leaf area (cm^2^). Results were expressed as mmol H_2_O cm^−2^ h^−1^.

### 4.7. Morphological Traits

Morphological traits were recorded at the flowering and podding stages from five randomly selected plants per genotype per treatment. Plant height was measured from the soil surface to the tip of the apical meristem of the main stem using a graduated ruler, and expressed in centimeters. Leaf number was determined by counting all fully expanded leaves on the main stem at the time of measurement. Node number was recorded as the total number of nodes on the main stem. Mean values across five plants were used for statistical analysis

### 4.8. Yield-Related Traits

Yield components were assessed at the harvest stage from all harvestable plants per genotype per treatment under lysimeter conditions. Pod number per plant was determined by counting all mature pods on each individual plant. Pod length was measured on ten randomly selected mature pods per plant using a ruler, and results were expressed in centimetres. Total grain weight per plant (g) was determined by weighing all seeds harvested from each individual plant on an analytical balance. 1000-seed weight (g) was determined by counting and weighing three sub-samples of 100 seeds from the pooled harvest of each genotype × treatment combination and multiplying by ten.

### 4.9. Statistical Analyses

All statistical analyses were performed in R (version 4.6.0; R Core Team, 2026, R Foundation for Statistical Computing, Vienna, Austria). The experimental unit was defined as an individual plant. Biological replicates consisted of three independently grown plants per genotype × treatment combination for hormonal, physiological, and morphological traits assessed at the budding, flowering, and podding stages, and all surviving plants per genotype × treatment combination (*n* = 5–18) for yield-related traits at harvest, reflecting natural variation in plant survival and pod set under infection.

For traits measured at the budding, flowering, and podding stages, two-way analysis of variance (ANOVA, Type I) was used to assess the main effects of genotype (G), treatment (T), and their interaction (G × T), allowing the relative contributions of genetic background, pathogen exposure, and their interaction to be assessed within a single model. For yield traits at the harvest stage, Type III ANOVA was applied to account for the unbalanced design arising from differential plant survival and pod set. Where a significant effect was detected (*p* < 0.05), Tukey’s honestly significant difference (HSD) test was used for pairwise comparisons among genotype × treatment means, implemented with the rstatix and agricolae packages; for the unbalanced harvest data, Tukey’s HSD was applied using the harmonic mean of subgroup sizes. For multivariate analyses, trait values were aggregated to genotype × treatment means (*n* = 18 combinations) and subjected to: principal component analysis (PCA) using the FactoMineR package, with variables standardised to zero mean and unit variance prior to analysis to prevent traits with larger numerical ranges from disproportionately influencing the ordination; Pearson correlation analysis using the GGally package, conducted separately for Control and Inoculated plants to detect pathogen-induced changes in inter-trait relationships; and hierarchical clustering using Ward’s minimum variance linkage method with Euclidean distance, implemented in the pheatmap package, conducted separately within each treatment group to characterise genotype similarity structures. K-means clustering (k = 3) was applied to PC1–PC2 scores to classify genotype × treatment profiles into functionally distinct groups. Statistical significance was set at α = 0.05 throughout.

## 5. Conclusions

This study evaluated nine mung bean genotypes under *A. alternata* infection across four growth stages, producing hormonal, physiological, morphological, and yield data with direct relevance for breeding disease-tolerant varieties.

Firstly, distinct hormonal strategies did not guarantee yield protection. Neither massive SA induction (Barqaror) nor constitutive SA/JA priming (Baraka, Turon) alone prevented substantial yield loss. Effective field resistance therefore depends on coordinated defence activity rather than a single hormonal response.

Secondly, the non-significant impact of infection on 1000-seed weight shows that A. alternata mainly targets pod initiation rather than seed filling. Breeding efforts should therefore prioritise floral and early reproductive development.

Thirdly, Zilola was the most agronomically promising genotype, combining near-complete pod retention (−0.8%), moderate grain weight loss (−26.7%), and a commercially acceptable seed size (5.85 → 5.32 g).

Finally, the marked shift in phenotypic relationships under infection confirms that baseline vigour alone does not predict stress tolerance. These conclusions rest on physiological and biochemical evidence and still need molecular confirmation. Future work should profile defence gene expression, including PR proteins and PAL, alongside ROS-scavenging enzyme activity. Metabolomic analysis and pathogen biomass quantification (e.g., qPCR) would further clarify these mechanisms. As only nine genotypes were evaluated here, these patterns will need testing in a wider range of mung bean germplasm before they can guide breeding programmes more broadly.

## Figures and Tables

**Table 1:** Chlorophyll *a*, chlorophyll *b*, total chlorophyll, and carotenoid contents (mg g^−1^ FW) of nine mung bean (*Vigna radiata* L.) genotypes under Control and *A. alternata*-inoculated conditions at the Flowering stage.

Genotype	Chl *a* (mg g^−1^ FW)		Chl *b* (mg g^−1^ FW)		Total Chl (mg g^−1^ FW)		Carotenoids (mg g^−1^ FW)	
	Control	Inoculated	Control	Inoculated	Control	Inoculated	Control	Inoculated
*Ishonch*	1.64 ± 0.25 abcde	1.20 ± 0.02 ef	0.49 ± 0.19 a	0.48 ± 0.02 a	2.46 ± 0.15 abcd	1.68 ± 0.04 gh	0.65 ± 0.04 abcd	0.47 ± 0.01 ef
*Turon*	1.59 ± 0.11 abcde	1.57 ± 0.03 abcde	0.57 ± 0.03 a	0.56 ± 0.02 a	2.15 ± 0.13 bcdefg	2.13 ± 0.04 bcdefg	0.67 ± 0.04 abcd	0.60 ± 0.01 cde
*Marjon*	1.63 ± 0.05 abcde	1.36 ± 0.02 def	0.58 ± 0.00 a	0.53 ± 0.01 a	2.21 ± 0.06 abcdef	1.88 ± 0.02 efgh	0.67 ± 0.04 abcd	0.55 ± 0.01 def
*Zamin*	1.98 ± 0.02 a	1.42 ± 0.05 cdef	0.69 ± 0.01 a	0.54 ± 0.02 a	2.68 ± 0.03 a	1.96 ± 0.07 efgh	0.76 ± 0.00 ab	0.54 ± 0.02 def
*Barqaror*	1.93 ± 0.03 ab	1.83 ± 0.07 abc	0.68 ± 0.01 a	0.65 ± 0.03 a	2.61 ± 0.03 ab	2.48 ± 0.10 abcd	0.78 ± 0.03 a	0.73 ± 0.03 abc
*Andijon-1*	1.70 ± 0.12 abcd	1.55 ± 0.06 abcde	0.62 ± 0.06 a	0.55 ± 0.03 a	2.31 ± 0.18 abcde	2.09 ± 0.09 cdefg	0.72 ± 0.06 abc	0.61 ± 0.02 bcde
*Baraka*	1.63 ± 0.01 abcde	1.05 ± 0.01 f	0.57 ± 0.01 a	0.44 ± 0.01 a	2.21 ± 0.01 abcdef	1.49 ± 0.01 h	0.61 ± 0.01 cde	0.41 ± 0.01 f
*Zilola*	1.89 ± 0.16 ab	1.25 ± 0.03 def	0.69 ± 0.06 a	0.49 ± 0.01 a	2.58 ± 0.22 abc	1.74 ± 0.02 fgh	0.71 ± 0.06 abc	0.48 ± 0.02 ef
*Durdona*	1.48 ± 0.02 bcdef	1.37 ± 0.03 def	0.53 ± 0.00 a	0.52 ± 0.02 a	2.00 ± 0.01 defg	1.89 ± 0.04 efgh	0.59 ± 0.01 cde	0.54 ± 0.01 def
Genotype (G)	F = 7.288, *p* < 0.001		F = 2.355, *p* = 0.038		—		F = 13.534, *p* < 0.001	
Treatment (T)	F = 61.475, *p* < 0.001		F = 8.903, *p* = 0.005		—		F = 107.322, *p* < 0.001	
G × T	F = 3.827, *p* = 0.002		ns		—		F = 3.375, *p* = 0.005	

Values are mean ± SE (*n* = 3 biological replicates). Different lowercase letters within a column indicate significant differences among genotype × treatment combinations (Tukey’s HSD, *p* < 0.05). Chl, chlorophyll; FW, fresh weight; ns, not significant.

**Table 2:** Chlorophyll *a*, chlorophyll *b*, total chlorophyll, and carotenoid contents (mg g^−1^ FW) of nine mung bean (*Vigna radiata* L.) genotypes under Control and *A. alternata*-inoculated conditions at the Podding stage.

Genotype	Chl *a* (mg g^−1^ FW)		Chl *b* (mg g^−1^ FW)		Total Chl (mg g^−1^ FW)		Carotenoids (mg g^−1^ FW)	
	Control	Inoculated	Control	Inoculated	Control	Inoculated	Control	Inoculated
*Ishonch*	1.62 ± 0.01 cd	0.90 ± 0.08 ef	0.59 ± 0.02 abcdefg	0.39 ± 0.05 defg	2.22 ± 0.03 bc	1.29 ± 0.12 de	0.43 ± 0.16 def	0.37 ± 0.05 ef
*Turon*	1.67 ± 0.09 bcd	1.95 ± 0.10 abc	0.65 ± 0.04 abcde	0.66 ± 0.02 abcd	2.32 ± 0.13 abc	2.62 ± 0.12 abc	0.62 ± 0.04 bcde	0.78 ± 0.04 abc
*Marjon*	1.67 ± 0.26 bcd	1.29 ± 0.02 de	0.61 ± 0.09 abcdef	0.49 ± 0.01 bcdefg	2.28 ± 0.35 abc	1.78 ± 0.03 cd	0.64 ± 0.11 bcde	0.51 ± 0.01 cdef
*Zamin*	2.15 ± 0.09 abc	2.23 ± 0.02 ab	0.75 ± 0.03 ab	0.89 ± 0.06 a	2.90 ± 0.12 ab	3.12 ± 0.07 a	0.82 ± 0.04 ab	0.86 ± 0.01 ab
*Barqaror*	2.27 ± 0.06 a	0.92 ± 0.02 ef	0.81 ± 0.04 a	0.39 ± 0.01 defg	3.08 ± 0.10 a	1.32 ± 0.04 de	0.95 ± 0.04 a	0.39 ± 0.01 ef
*Andijon-1*	1.65 ± 0.04 cd	0.87 ± 0.06 ef	0.64 ± 0.03 abcde	0.44 ± 0.03 cdefg	2.29 ± 0.07 abc	1.30 ± 0.08 de	0.64 ± 0.03 bcde	0.37 ± 0.02 ef
*Baraka*	1.79 ± 0.07 abcd	0.59 ± 0.01 f	0.65 ± 0.03 abcde	0.30 ± 0.01 g	2.43 ± 0.10 abc	0.89 ± 0.02 e	0.66 ± 0.02 abcde	0.28 ± 0.01 f
*Zilola*	1.87 ± 0.10 abc	0.75 ± 0.11 ef	0.72 ± 0.04 abc	0.35 ± 0.03 efg	2.59 ± 0.14 abc	1.10 ± 0.14 de	0.71 ± 0.04 abcd	0.32 ± 0.03 f
*Durdona*	1.94 ± 0.26 abc	0.76 ± 0.06 ef	0.66 ± 0.19 abcd	0.31 ± 0.03 fg	2.60 ± 0.45 abc	1.07 ± 0.09 de	0.73 ± 0.09 abc	0.30 ± 0.02 f
Genotype (G)	F = 18.406, *p* < 0.001		F = 7.048, *p* < 0.001		—		F = 11.315, *p* < 0.001	
Treatment (T)	F = 194.275, *p* < 0.001		F = 56.100, *p* < 0.001		—		F = 68.886, *p* < 0.001	
G × T	F = 14.934, *p* < 0.001		F = 5.174, *p* < 0.001		—		F = 8.730, *p* < 0.001	

Values are mean ± SE (*n* = 3 biological replicates). Different lowercase letters within a column indicate significant differences among genotype × treatment combinations (Tukey’s HSD, *p* < 0.05). Chl, chlorophyll; FW, fresh weight; ns, not significant.

**Table 3:** Plant height (cm), leaf number, and node number of nine mung bean (*Vigna radiata* L.) genotypes under Control and *A. alternata*-inoculated conditions at the Flowering stage.

Genotype	Plant Height (cm)		Leaf Number		Node Number	
	Control	Inoculated	Control	Inoculated	Control	Inoculated
*Ishonch*	29.00 ± 2.08 bcde	24.33 ± 0.33 de	9.33 ± 1.86 abc	11.33 ± 1.76 abc	7.67 ± 0.88 abcde	6.67 ± 0.33 bcde
*Turon*	29.00 ± 0.58 bcde	29.67 ± 2.33 abcde	11.33 ± 0.67 abc	11.33 ± 2.96 abc	7.67 ± 0.33 abcde	8.33 ± 0.33 abc
*Marjon*	28.33 ± 1.86 cde	37.67 ± 2.60 ab	12.67 ± 1.86 abc	13.00 ± 2.89 abc	7.33 ± 0.67 bcde	8.67 ± 0.33 ab
*Zamin*	31.67 ± 1.86 abcde	34.33 ± 1.76 abc	9.33 ± 1.86 abc	19.33 ± 2.40 a	6.00 ± 0.58 bcde	8.00 ± 1.15 abcd
*Barqaror*	32.00 ± 0.58 abcde	23.67 ± 2.85 e	7.33 ± 0.33 bc	12.00 ± 3.06 abc	5.33 ± 0.33 bcde	11.00 ± 1.15 a
*Andijon-1*	26.67 ± 1.33 cde	38.33 ± 0.88 a	7.33 ± 0.67 bc	17.33 ± 0.88 ab	5.33 ± 0.33 bcde	11.00 ± 0.58 a
*Baraka*	24.00 ± 1.00 e	25.67 ± 0.88 cde	7.67 ± 1.20 bc	18.33 ± 3.33 a	4.67 ± 0.33 de	8.00 ± 0.00 abcd
*Zilola*	24.67 ± 0.88 de	31.00 ± 0.58 abcde	6.67 ± 0.33 c	11.00 ± 2.65 abc	4.33 ± 0.33 e	7.33 ± 1.20 bcde
*Durdona*	24.67 ± 0.88 de	33.33 ± 3.53 abcd	7.00 ± 0.00 c	11.00 ± 0.00 abc	5.00 ± 0.00 cde	7.33 ± 0.67 bcde
Genotype (G)	F = 5.729, *p* < 0.001		F = 2.044, *p* = 0.069		F = 4.220, *p* = 0.001	
Treatment (T)	F = 14.489, *p* < 0.001		F = 31.530, *p* < 0.001		F = 72.136, *p* < 0.001	
G × T	F = 7.337, *p* < 0.001		F = 2.336, *p* = 0.039		F = 5.864, *p* < 0.001	

Values are mean ± SE (*n* = 3 biological replicates). Different lowercase letters within a column indicate significant differences among genotype × treatment combinations (Tukey’s HSD, *p* < 0.05). ns, not significant.

**Table 4:** Plant height (cm), leaf number, and node number of nine mung bean (*Vigna radiata* L.) genotypes under Control and *A. alternata*-inoculated conditions at the Podding stage.

Genotype	Plant Height (cm)		Leaf Number		Node Number	
	Control	Inoculated	Control	Inoculated	Control	Inoculated
*Ishonch*	41.00 ± 4.62 bcdef	29.33 ± 0.33 fg	12.33 ± 2.85 d	10.67 ± 1.33 d	8.33 ± 0.67 abc	6.67 ± 0.33 c
*Turon*	45.33 ± 2.91 abcd	40.00 ± 1.53 cdef	16.00 ± 0.58 bcd	15.33 ± 2.19 bcd	8.67 ± 0.33 abc	8.33 ± 0.33 abc
*Marjon*	49.00 ± 1.53 abcd	41.33 ± 2.19 bcdef	18.00 ± 3.06 bcd	20.33 ± 1.86 bcd	13.33 ± 2.73 a	8.67 ± 0.33 abc
*Zamin*	41.67 ± 0.88 bcdef	41.33 ± 2.91 bcdef	17.00 ± 0.58 bcd	16.67 ± 3.53 bcd	13.00 ± 0.58 ab	8.00 ± 1.15 abc
*Barqaror*	55.33 ± 2.03 a	51.67 ± 3.28 abc	25.67 ± 2.40 ab	24.67 ± 0.67 abc	13.00 ± 2.31 ab	11.00 ± 1.15 abc
*Andijon-1*	53.00 ± 3.00 ab	52.00 ± 1.15 abc	23.33 ± 0.88 abc	20.00 ± 1.00 bcd	10.33 ± 0.88 abc	11.00 ± 0.58 abc
*Baraka*	37.67 ± 2.91 defg	25.67 ± 0.88 g	32.67 ± 0.33 a	16.67 ± 3.18 bcd	8.33 ± 0.33 abc	8.00 ± 0.00 abc
*Zilola*	31.33 ± 1.86 efg	26.00 ± 2.08 g	17.33 ± 1.76 bcd	15.00 ± 1.53 cd	8.00 ± 0.00 abc	7.33 ± 1.20 bc
*Durdona*	42.33 ± 1.76 bcde	25.67 ± 3.28 g	19.33 ± 2.73 bcd	14.33 ± 0.67 cd	10.00 ± 1.00 abc	7.33 ± 0.67 bc
Genotype (G)	F = 27.052, *p* < 0.001		F = 10.036, *p* < 0.001		F = 4.682, *p* < 0.001	
Treatment (T)	F = 38.727, *p* < 0.001		F = 10.872, *p* = 0.002		F = 13.369, *p* < 0.001	
G × T	F = 2.555, *p* = 0.026		F = 3.436, *p* = 0.005		ns	

Values are mean ± SE (*n* = 3 biological replicates). Different lowercase letters within a column indicate significant differences among genotype × treatment combinations (Tukey’s HSD, *p* < 0.05). ns, not significant.

**Table 5:** Pod number, pod length (cm), grain weight (g), and 1000-seed weight (g) of nine mung bean (*Vigna radiata* L.) genotypes under Control and *A. alternata*-inoculated conditions at the Harvest stage.

Genotype	Pod Number		Pod Length (cm)		Grain Weight (g)		1000-Seed Weight (g)	
	Control	Inoculated	Control	Inoculated	Control	Inoculated	Control	Inoculated
*Ishonch*	59.82 ± 8.21 ab	43.50 ± 6.04 abcd	11.15 ± 0.52 ab	7.95 ± 0.12 de	30.44 ± 4.27 abc	13.23 ± 2.05 c	5.68 ± 0.14 cd	5.98 ± 0.46 bcd
*Turon*	59.33 ± 6.15 abc	32.18 ± 5.42 bcd	10.17 ± 0.36 abcd	8.81 ± 0.32 cde	31.01 ± 3.46 abc	17.19 ± 3.38 bc	6.27 ± 0.09 abcd	6.17 ± 0.24 bcd
*Marjon*	56.67 ± 7.60 abc	37.11 ± 2.48 abcd	9.33 ± 0.19 bcd	7.75 ± 0.37 de	32.15 ± 4.08 ab	17.27 ± 1.22 bc	6.08 ± 0.10 bcd	5.64 ± 0.14 cd
*Zamin*	45.17 ± 4.92 abcd	24.54 ± 2.26 d	11.30 ± 0.40 ab	9.40 ± 0.19 bcd	30.18 ± 3.34 abc	12.18 ± 1.46 c	7.18 ± 0.19 ab	6.76 ± 0.38 abc
*Barqaror*	70.60 ± 11.12 a	33.22 ± 4.68 abcd	9.30 ± 0.34 bcde	8.61 ± 0.53 cde	32.06 ± 4.80 abc	12.00 ± 2.75 c	6.11 ± 0.20 bcd	6.21 ± 0.19 bcd
*Andijon-1*	68.60 ± 7.91 a	31.56 ± 3.97 cd	10.90 ± 0.37 ab	9.80 ± 0.46 abcd	40.73 ± 5.34 a	18.23 ± 2.52 bc	7.77 ± 0.17 a	7.78 ± 0.13 a
*Baraka*	51.88 ± 6.37 abcd	47.20 ± 4.55 abcd	9.94 ± 1.05 abcd	6.60 ± 0.48 e	15.33 ± 2.69 bc	11.74 ± 1.97 c	3.58 ± 0.17 e	3.03 ± 0.31 e
*Zilola*	44.19 ± 5.70 abcd	43.85 ± 5.27 abcd	11.55 ± 0.67 a	8.50 ± 0.21 cde	21.54 ± 2.71 bc	15.78 ± 2.19 bc	5.85 ± 0.32 cd	5.32 ± 0.37 d
*Durdona*	51.54 ± 5.89 abcd	34.10 ± 6.12 abcd	11.70 ± 0.31 a	10.20 ± 0.23 abc	22.31 ± 2.38 bc	12.09 ± 2.43 c	5.19 ± 0.24 d	5.17 ± 0.37 d
Genotype (G)	F = 1.871, *p* = 0.067		F = 7.855, *p* < 0.001		F = 3.930, *p* < 0.001		F = 34.615, *p* < 0.001	
Treatment (T)	F = 45.284, *p* < 0.001		F = 85.864, *p* < 0.001		F = 74.051, *p* < 0.001		F = 1.780, *p* = 0.184	
G × T	F = 2.213, *p* = 0.028		F = 2.267, *p* = 0.026		F = 1.847, *p* = 0.071		F = 0.611, *p* = 0.768	

Values are mean ± SE (*n* = 5–18 biological replicates per genotype × treatment combination—unbalanced design). Different lowercase letters within a column indicate significant differences among genotype × treatment combinations (Tukey’s HSD, *p* < 0.05). Tukey HSD was applied using the harmonic mean of replicate numbers for unbalanced groups. ns, not significant (*p* ≥ 0.05).

## Data Availability

The data presented in this study are available from the corresponding author upon reasonable request.

## Supplementary Materials

The following supporting information can be downloaded at https://www.mdpi.com/article/10.3390/plants15152397/s1, Figure S1. Pearson correlation matrices of 16 measured traits in nine mung bean (*Vigna radiata* L.) genotypes under (A) Control and (B) *Alternaria alternata*-inoculated conditions. Colour gradient indicates Pearson correlation coefficient (r) from −1 (blue) to +1 (red). Asterisks denote statistical significance: * *p* < 0.05; ** *p* < 0.01; *** *p* < 0.001.
