# Supplementary material for: Integrated Physiological and Biochemical Responses of Mungbean (Vigna radiata) to Alternaria alternata
Source: Plants (Basel). 2026 Aug 5;15(15):2397. doi: 10.3390/plants15152397 (PMC13467819; doi:10.3390/plants15152397)

Supplementary Materials

Figure S1. Pearson correlation matrices of 16 measured traits in nine mung bean (Vigna radiata L.) genotypes under (A) Control and (B) Alternaria alternata-inoculated conditions. Colour gradient indicates Pearson correlation coefficient (r) from −1 (blue) to +1 (red). Asterisks denote statistical significance: * $p < 0.05$; ** $p < 0.01$; *** $p < 0.001$.

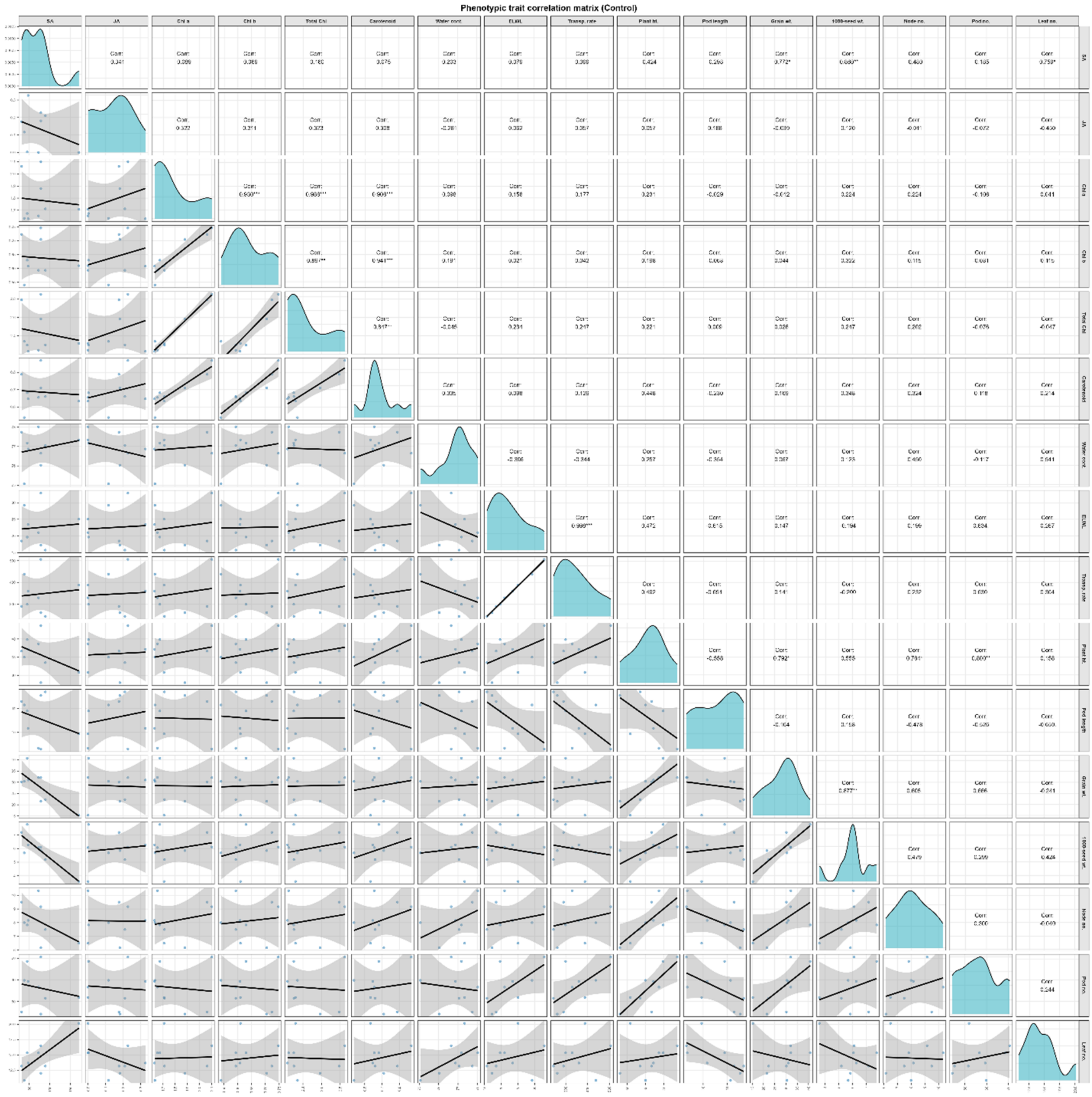

Phenotypic trait correlation matrix (Inoculated)

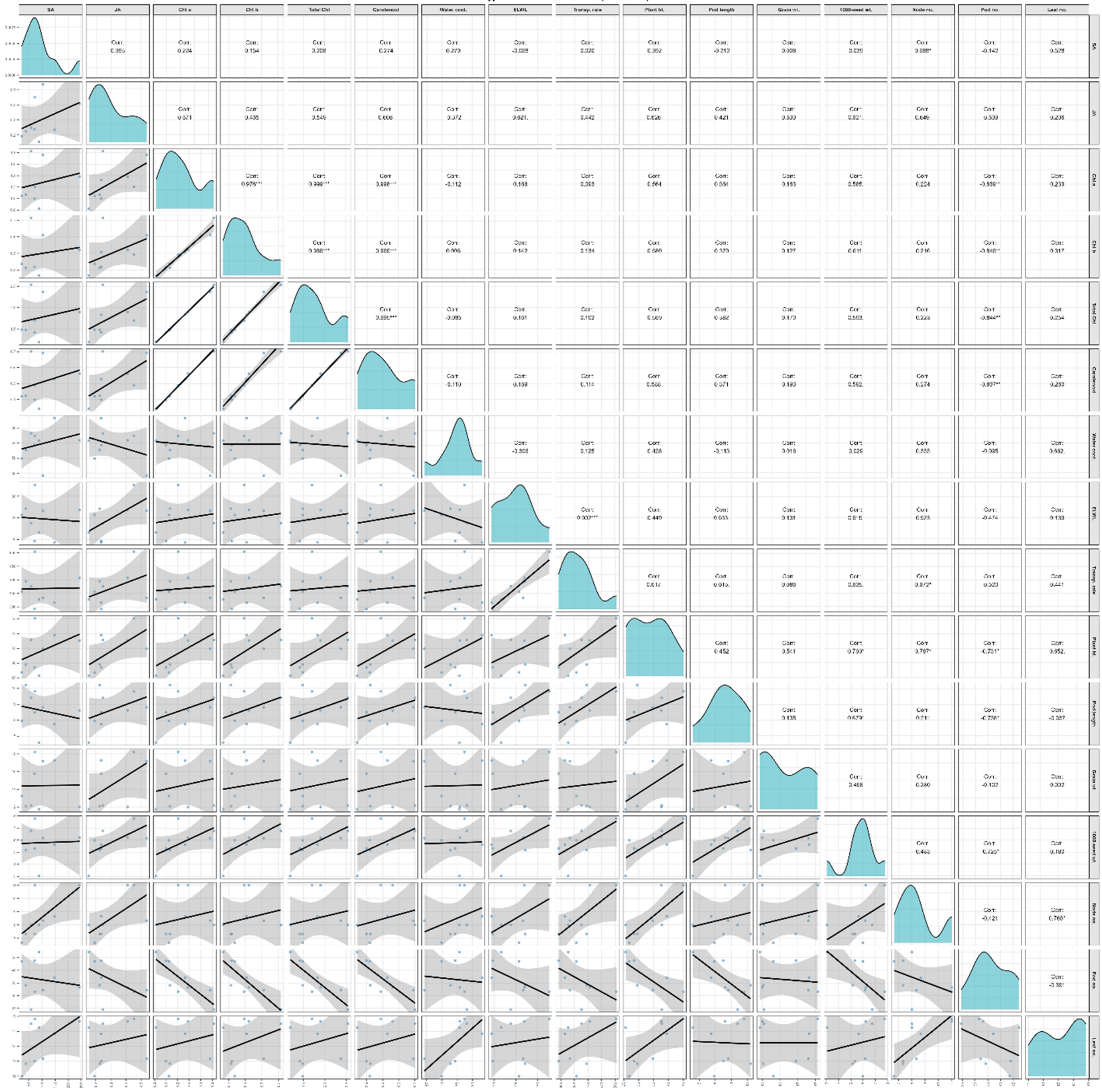

Supplement: Supplementary file 1 [file plants-15-02397-s001.zip › plants-4453764-supplementary.pdf]
